# Whole grain and refined grain consumption and the risk of hypertension: a systematic review and meta-analysis of prospective studies

**DOI:** 10.1038/s41598-025-05197-5

**Published:** 2025-07-01

**Authors:** Dagfinn Aune, Michael Metoudi, Isabelle Sadler, Shireen Kassam

**Affiliations:** 1 Department of Epidemiology and Biostatistics, School of Public Health, Imperial College London, London, UK; 2 Department of Research, The Cancer Registry of Norway, Norwegian Institute of Public Health, Oslo, Norway; 3 Department of Nutrition, Oslo New University College, Oslo, Norway; 4 Plant-Based Health Professionals, London, UK; 5 King’s College London and King’s College Hospital, London, UK; 6 University of Winchester, Hampshire, UK

**Keywords:** Whole grains, Refined grains, Hypertension, Cohort, Meta-analysis, Hypertension, Risk factors

## Abstract

A high intake of whole grains has been associated with a reduced risk of hypertension, however, studies have not been entirely consistent. Findings regarding refined grains and hypertension have also been inconsistent. We conducted a systematic review and meta-analysis of prospective cohort studies on whole grain and refined grain consumption and hypertension risk. PubMed and Embase databases were searched up to 25th of July 2024. Random effects models were used to estimate summary relative risks (RRs) and 95% confidence intervals (CIs) for the association between whole grain and refined grain intake and hypertension. Restricted cubic splines were used to investigate potential nonlinear associations. Nine cohort studies were included in the meta-analysis. The summary RR (95% CI) for high vs. low whole grain intake was 0.74 (0.59–0.93, I^2^ = 97%, p_heterogeneity_<0.001, *n* = 9) and per 90 g/d was 0.86 (0.82–0.90, I^2^ = 63%, p_heterogeneity_=0.008, *n* = 8). The summary RR (95% CI) for high vs. low refined grain intake was 0.94 (0.88–1.01, I^2^ = 7.9%, p_heterogeneity_=0.36, *n* = 5) and per 90 g/d was 0.97 (0.93–1.02, I^2^ = 0%, p_heterogeneity_=0.42, *n* = 4). There was no indication of publication bias in either analysis, although the number of studies was low for refined grains. There was no evidence of nonlinearity for whole grains (p_nonlinearity_=0.31) or refined grains (p_nonlinearity_=0.21), and for whole grains there was a 22% reduction in risk at 200 vs. 0 g/d. These findings provide further support for a beneficial role of whole grain consumption in relation to hypertension risk and support recommendations to increase whole grain intake in the general population. No clear association was observed between refined grains and risk of hypertension.

**Supplementary Information:**

The online version contains supplementary material available at 10.1038/s41598-025-05197-5.

## Introduction

Globally the number of people with hypertension doubled between 1990 and 2019 from 648 million to 1.28 billion^1^. Hypertension is a major risk factor for multiple cardiovascular outcomes^2–7^ and high systolic blood pressure is the leading and second leading risk factor for premature mortality and loss of disability-adjusted life years (DALYs) worldwide, accounting for 10.9 million deaths and 7.8% of total DALYs in 2021, respectively, according to the Global Burden of Disease Study^8^.

A high whole grain intake has been associated with reduced risk of cardiovascular disease^9^, all-cause mortality^9^, and other health outcomes^10,11^. Although studies have been quite consistent in showing cardiovascular benefits with a high whole grain intake, the underlying pathways or mechanisms that explain these benefits need clarification. Whole grains are high in fiber, which may reduce weight gain and risk of obesity^12^, which again is strongly associated with increased risk of hypertension^13^. Several studies have been published on whole grain consumption and hypertension risk^14–22^, and most of these suggested a high whole grain intake is associated with a lower risk of hypertension^14–17,19,21^, although two studies reported no clear association^18,20^. In the CARDIA study (4304 participants, 997 cases), the highest vs. lowest quintile of whole grain consumption (> 1.9 vs. <0.4 times/day) was associated with a 17% reduced risk of hypertension^14^. In the Women’s Health Study (28926 participants, 8722 cases), the highest vs. lowest quintile of whole grain consumption (3.07 vs. 0.21 servings/day) was associated with an 11% reduction in risk of hypertension^15^. In the Health Professionals Follow-up Study (31684 participants, 9227 cases), the highest vs. lowest quintile of whole grain intake (46.0 vs. 3.3 g/d) was associated with a 19% reduction in hypertension risk^16^. In the Physicians’ Health Study I (13368 participants, 7267 cases), the highest vs. lowest category (≥ 7 vs. 0 servings/week) of whole grain cereal consumption was associated with a 20% reduction in risk of hypertension. In the NutriNet-Sante study (80426 participants, 2413 cases), the highest vs. lowest category (102 vs. 0 g/d) of whole grain consumption was associated with a 16% reduction in risk of hypertension. In the Furukawa Nutrition and Health Study (944 participants, 86 cases) the highest vs. lowest category of whole grain intake (sometimes or always vs. never) was associated with a 64% reduction in the risk of hypertension and in the China Health and Nutrition Survey a 65% reduction in hypertension risk was observed for those in the highest vs. lowest quartile of whole grain consumption^22^. However, both the CoLaus Study (2079 participants, 370 cases) and the Atherosclerosis Risk in Communities Study (9913 participants, 1663 cases) reported no clear association between whole grain intake and hypertension risk^18,20^.

Studies on refined grain consumption have been less consistent and have in general shown no clear association^14,15,17,18,20,23^, however, a Chinese study reported an inverse association between intake of rice and risk of hypertension^23^, and another study reported a borderline significant inverse association with refined grains^17^ and two other studies reported non-significant inverse associations^14,20^, but may have been underpowered to detect a clear association. We conducted a systematic review and meta-analysis of prospective studies to clarify the association between whole grain and refined grain intake and hypertension risk.

## Methods

### Search strategy

PubMed and Embase databases were searched from inception to July 2023 for relevant studies and later updated to 25th of July 2024. The search strategy is provided in the Supplementary Text and included terms for other foods as part of a larger project. For the purposes of this review, the current analysis focused on whole grains and refined grains. We followed PRISMA criteria for reporting meta-analyses^24^. We also searched the reference lists of the included studies and of previous meta-analyses and reviews to identify any further and potentially relevant studies.

### Inclusion criteria and study selection

Prospective cohort studies, nested case-control studies within cohort studies and case-cohort studies that reported adjusted relative risk estimates for the association between whole grain or refined grain intake and risk of hypertension or elevated blood pressure were eligible for inclusion. Studies on blood pressure as a continuous variable were excluded. Retrospective case-control studies, cross-sectional studies, conference abstracts and grey literature were not included. A list of the excluded studies can be found in Supplementary Table 1. Two reviewers (MM and IS) independently evaluated each study in duplicate for their relevance in line with the inclusion criteria. Any outstanding disagreements of selected studies were further resolved by discussion with a third reviewer (DA).

### Data extraction

The following data were extracted from each study: first author’s last name, publication year, country where the study was conducted, study period, number of participants and cases, age, sex, exposure, quantity, relative risk (RR) estimates and 95% confidence intervals (CIs), and variables adjusted for in the analyses. One reviewer (MM) extracted the relevant data and it was further checked for accuracy by a second reviewer (DA).

### Assessment of study quality

Two authors (MM and IS) independently assessed all included studies for their respective quality using a modified version of the Newcastle Ottowa Scale as described previously^25^, which categories a scoring domain into low (0–3), medium (> 3–6), and high (> 6–8) quality respectively. Any discrepancies between the two reviewers were discussed and resolved with a third reviewer (DA).

### Assessment of strength of evidence

We used the grading criteria of the World Cancer Research Fund to assess the strength of evidence^26,27^. The grading system incorporates a range of factors including evidence from different study types, the number of studies available, heterogeneity, quality of the studies, dose-response relationship, biological plausibility, and experimental evidence. Evidence is graded as (1) substantial effect on risk unlikely, (2) limited-no conclusion, (3) limited-suggestive, (4) probable and (5) convincing evidence of a causal relationship using specific criteria^26,27^.

### Statistical methods

We used the random effects model by DerSimonian and Laird^28^, which takes into account both within and between study heterogeneity, to calculate summary RRs (95% CIs) for the association between whole grain and refined grain intake and hypertension risk. The average of the natural logarithm of the RRs was estimated and the RR from each study was weighted using random effect weights. Linear dose-response analyses were conducted using the method by Greenland and Longnecker^29^. For studies that reported whole grain or refined grain intake by ranges of intake we estimated the midpoint for each category by calculating the average of the lower and higher cut-off points. When extreme categories were open-ended, we used the width of the adjacent interval to estimate a lower or higher cut-off point. For studies that reported intakes in servings/day or times/day, we converted the quantities to grams/day by using 30 g as a serving size (equal to one slice of whole grain bread)^9^, while for one study that reported on intake of breakfast cereals^17^ we used a serving size of 90 g. For one paper which did not report quantities of whole grain and refined grain intake per quintile^18^, we used the quantities reported in another paper from the same study (also categorized by quintiles)^30^, and for another study we obtained supplementary information on quantities of whole grain intake from the authors^20^. We used restricted cubic splines to investigate potential nonlinear associations, with three knots at 10%, 50% and 90% centiles of the distribution which were combined using multivariable meta-analysis^31^.

Heterogeneity between studies was evaluated with I^2^-statistics^32^. We conducted subgroup and meta-regression analyses to investigate potential sources of heterogeneity between studies, including sex, duration of follow-up, geographic location, study quality, use of repeated dietary assessments over the follow-up period and adjustment for confounding factors. Sensitivity analyses were conducted to investigate the robustness of the findings by excluding one study at a time from the meta-analysis to test whether the results were driven by one very large or outlying study. We used E-values to estimates the strength of an unadjusted confounder that could explain away the observed associations^33^. The E-value is defined as the minimum strength and unmeasured or uncontrolled confounder would have with both the exposure and the outcome to fully explain away the observed associations. Publication bias was assessed using Egger’s test^34^ and by inspection of the funnel plots. The statistical analyses were conducted using STATA version 17.0 (StataCorp, College Station, TX, USA).

## Results

**Fig. 1:**
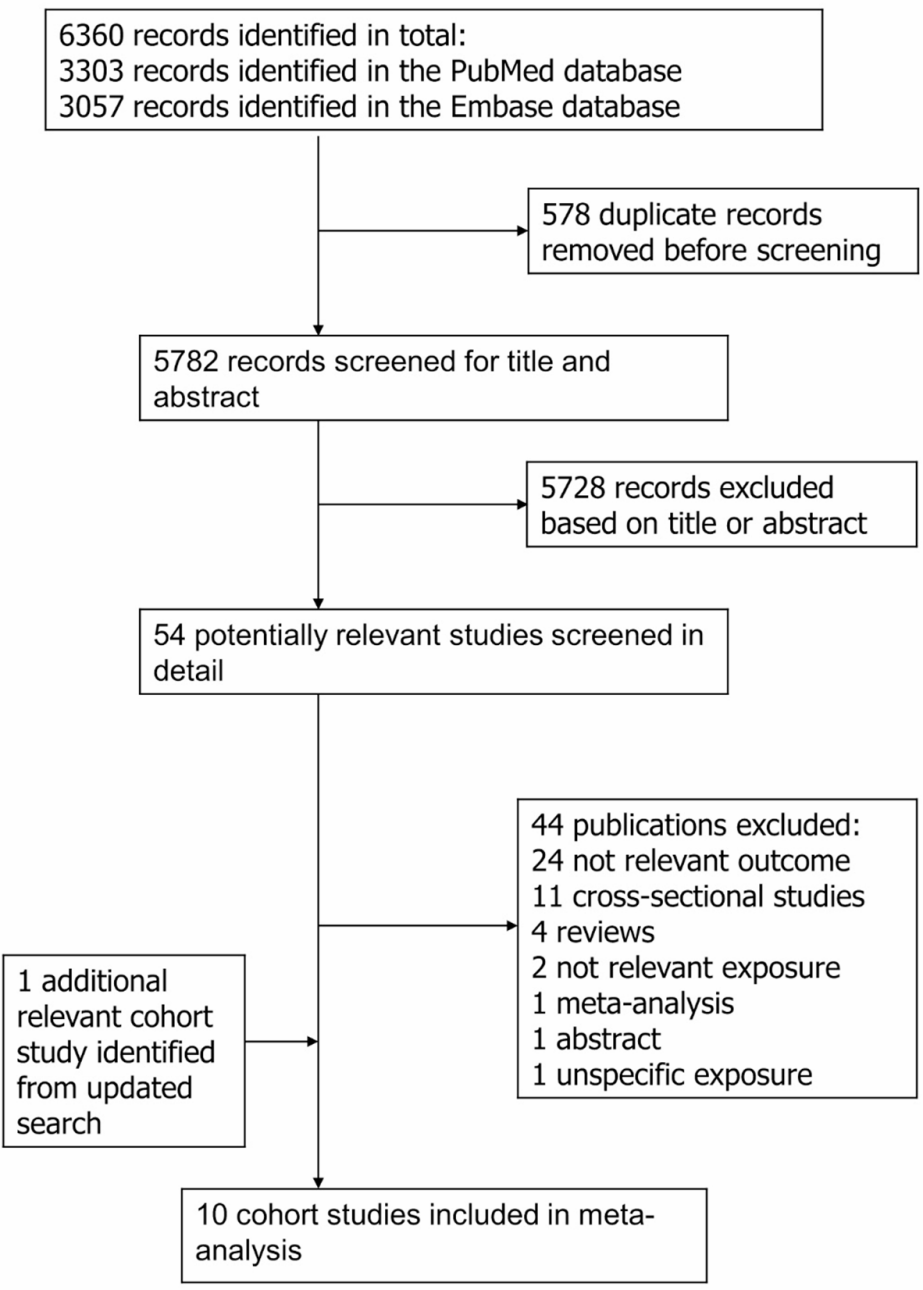
Flow-chart of study selection.

From a total of 6360 records that were screened, ten prospective cohort studies^14–23^ were included in the current systematic review and nine studies^14–22^ were included in the meta-analysis (Fig. 1). Only one study was published on intake of rice and hypertension risk and reported an inverse association, but could not be meta-analysed because of a lack of other studies^23^. The characteristics of the included studies are shown in Supplementary Table 2. Five of the studies were from the US, two were from Europe (Switzerland and France), and three were from Asia (one from Japan and two from China). Seven studies included both men and women, two studies included only men and one study included only women. The age range of the participants across the studies that reported this was 18-85.9 years. Information on how each study defined whole grains or refined grains is provided in Supplementary Table 3. The mean (median) study quality was 6.3 (6.8) across studies, with five studies having medium study quality and five studies having high study quality (Supplementary Table 4).

### Whole grains

**Fig. 2:**
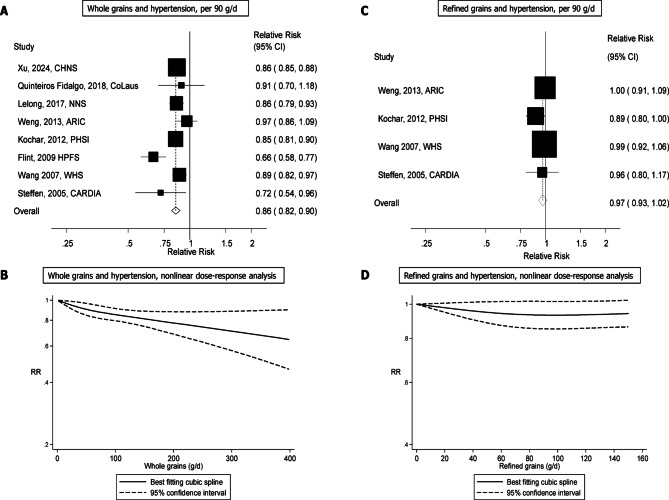
Whole grain and refined grain consumption and hypertension, linear (**A**, **C**) and nonlinear (**B**, **D**) dose-response analyses.

Nine studies (182617 participants, 33582 cases)^14–22^ were included in the high vs. low analysis and eight studies^14–20,22^were included in the dose-response analysis of whole grains and hypertension risk. The summary RR (95% CI) for high vs. low whole grain intake was 0.74 (0.59–0.93, I^2^ = 97%, p_heterogeneity_<0.001, *n* = 9) (Supplementary Fig. 1) and per 90 g/day was 0.86 (0.82–0.90, I^2^ = 63%, p_heterogeneity_=0.008, *n* = 8) (Fig. 2a). The summary RR ranged from 0.84 (0.81–0.89) when the study by Weng et al.^18^ was excluded to 0.87 (0.85–0.88) when the study by Flint et al.^16^ was excluded (Supplementary Fig. 2). There was no indication of publication bias with Egger’s test (*p* = 0.59), Begg’s test (*p* = 0.54) or by inspection of the funnel plot (Supplementary Fig. 3). There was no evidence of nonlinearity (p_nonlinearity_=0.31) (Fig. 2b, Supplementary Table 5).

### Refined grains

Five studies (58590 participants, 16074 cases)^14,15,17,18,20^ were included in both the high vs. low and the dose-response analyses of refined grain consumption and hypertension risk. The summary RR (95% CI) for high vs. low refined grain intake was 0.94 (0.88–1.01, I^2^ = 7.9%, p_heterogeneity_=0.36, *n* = 5) (Supplementary Fig. 4) and per 90 g/day was 0.96 (0.92–1.01, I^2^ = 7.8%, p_heterogeneity_=0.36, *n* = 5) (Fig. 2c). The summary RR ranged from 0.95 (0.89–1.03) when excluding the study by Wang et al.^15^ to 0.99 (0.94–1.04) when excluding the study by Kochar et al.^17^. There was no indication of publication bias with Egger’s test (*p* = 0.21), Begg’s test (*p* = 0.46), or by inspection of the funnel plot (Supplementary Fig. 5). There was no indication of nonlinearity for refined grains and the association was null across the range of intakes (p_nonlinearity_=0.14) (Fig. 2d, Supplementary Table 6).

### Subgroup and sensitivity analyses

The inverse association between whole grain intake and hypertension persisted across most subgroup analyses, although numbers were low in some subgroups and there was no evidence of heterogeneity between subgroups (Supplementary Table 7). The heterogeneity was lower in subgroups of studies with both men and women combined, European studies, and studies with high study quality, and additionally in studies with adjustment for education, and BMI (Supplementary Table 7). The estimated E-values (lower CI) for intakes of 100, 200 and 300 g/d were 1.63 (1.43), 1.88 (1.53), and 2.17 (1.50) (Supplementary Table 5).

### Assessment of strength of evidence

When using the World Cancer Research Fund criteria for evaluating evidence, we considered the overall evidence to be supportive of a probably causal relationship between whole grain intake and reduced risk of hypertension, but for refined grains the evidence was limited and no conclusion could be made. A justification of these judgements is found in Supplementary Tables 8 and include clear inverse associations between high whole grain intake and hypertension across high vs. low, linear and nonlinear dose-response analyses, moderate to high heterogeneity which is mainly driven by differences in the size (not the direction) of the associations, and consistency of the results in subgroup and sensitivity analyses. In addition, plausible mechanisms exist and there is some evidence from randomized trials that fiber intake, which whole grains are an important source of, can reduce blood pressure. For refined grains, no clear association was observed and fewer studies were available and it was therefore graded as limited-no conclusion.

## Discussion

In this meta-analysis of 9 cohort studies, we observed a 26% lower risk of hypertension for high vs. low intake of whole grains and a 14% lower risk per 90 g/day, which is equal to three slices of whole grain bread or one bowl of whole grain cereals per day. The association was dose-dependent and linear and there was a 22% reduction in risk at 200 g/day. Refined grain consumption was not clearly associated with risk of hypertension.

Our findings are consistent with a previous meta-analysis of four prospective studies on whole grain consumption and hypertension risk, which found a 14% reduction in risk for high vs. low intake and an 8% reduction in risk per 30 g/d^35^. The results are also consistent with studies on dietary patterns high in whole grains and changes in blood pressure or hypertension risk^36–38^, such as the DASH diet^36^, Mediterranean dietary pattern^37^, vegetarian diets^38,39^, and plant-based diets in general^40^, although these dietary patterns also often are characterized by other beneficial components that may lower blood pressure, such as high intake of fruits, vegetables, and nuts and less salt and red and processed meat.

Our meta-analysis has some limitations that need to be discussed. A high whole grain intake may be associated with a generally healthy lifestyle pattern that potentially could confound the observed association. However, major risk factors for hypertension, such as alcohol consumption, obesity, and physical activity, were adjusted for in the majority of the studies and the inverse association between whole grain intake and hypertension persisted in subgroups of studies that adjusted for these confounders. The estimated E-values for intakes of 100, 200, and 300 g/d of whole grains were 1.63 (1.43), 1.88 (1.53), and 2.17 (1.50), suggesting a relatively strong unadjusted confounder would be needed to fully explain away the observed association. The inverse association persisted across most subgroups with adjustment for dietary factors (e.g. fruits and vegetables, meat, dairy, energy intake), and there was no indication of between-subgroup heterogeneity. The heterogeneity in the high vs. low analysis was very high, but it was considerably lower in the dose-response analysis. However, the observed heterogeneity appears to be more due to differences in the size of the association rather than direction of the association, as all studies reported risk estimates in the direction of a reduced risk. The high heterogeneity in the high vs. low analysis seemed to be at least partly driven by a much higher whole grain intake and considerably stronger reductions in hypertension risk in a Chinese study^22^ than in the remaining studies, however, in the linear dose-response analysis, when intakes were standardized across studies, the study showed comparable reductions in risk to the remaining studies, and the heterogeneity was lower. In subgroup analyses, there was no indication of between-subgroup heterogeneity when analyses were stratified by sex, duration of follow-up, geographic location, number of cases, study quality, whether the dietary assessment was validated or not, and adjustment for a range of confounders, however, additional studies could clarify some of these findings further with sex-stratified analyses, and with data from other regions than North America, as the number of studies in these strata were low. Although we found no indication of publication bias with Egger’s test, it is still possible that publication bias could have affected the results because of the limited number of studies included, particularly for refined grains. Further studies are therefore warranted. Measurement errors in the assessment of whole grain and refined grain intake may have occurred, however, no studies corrected for measurement errors, while four studies used repeated dietary assessments to assess whole grain intake over the follow-up period. Although there was no significant heterogeneity between subgroups using baseline only or updated dietary data, the estimates from studies using updated dietary assessments appeared somewhat more pronounced. Additional studies are needed to investigate this further. It is also possible that we may have introduced some measurement errors by using standard serving sizes when converting data from studies only reporting on frequency of intake to grams/day, however, this is standard practice across different food groups^9,41,42^, and difficult to handle in any other way in dose-response meta-analyses of published studies. Strengths of the meta-analysis include the comprehensive search strategy and analyses, and the multiple subgroup and sensitivity analyses that were conducted.

Several biological mechanisms could explain a beneficial effect of whole grain consumption on risk of hypertension. Whole grains are high in dietary fiber that can improve insulin sensitivity, which is thought to play a role in endothelial dysfunction and hypertension^43^, and regulate blood glucose and is beneficial in reducing the development of obesity^44^, possibly through greater satiety^45^. Several randomized trials have shown dietary fiber^46^, and whole grains^47^ can improve endothelial function, while another study showed increased nitric oxide production among persons fed a beta-glucan rich oat bread, although no difference was observed in flow-mediated dilation^48^. A high versus low whole grain intake has been associated with a lower risk of obesity and weight gain in epidemiological studies^12^, and overweight and obesity is a strong risk factor for the development of hypertension^13^. In addition, there is evidence from a meta-analysis of randomized trials that dietary fiber can reduce systolic blood pressure^44^, and results for whole grains were in the same direction, but were less precise as they were based on fewer studies. Other meta-analyses have suggested oats or beta-glucan rich interventions reduces blood pressure^49^. Avenanthramide, an antioxidant found in oats, has been found to increase production of nitric oxide, which regulates endothelial function^50^. It is likely that impact of higher intakes of dietary fiber on the gut microbiome with greater production of short chain fatty acids may also be responsible for the blood pressure lowering effects of whole grains^51^, through receptors that regulate vascular tone and inflammation. Some whole grains such as quinoa, amaranth and oats have a higher content of potassium and magnesium which may contribute to blood pressure regulation^52,53^. Several randomized trials and meta-analyses have shown whole grains reduce markers of inflammation^54–56^, primarily in persons with cardiovascular risk factors, and there is growing evidence that inflammation plays a role in the development of hypertension^57^. A recent workplace intervention study from Nepal that emphasized increased intake of whole grains, unsaturated fat, fruits, vegetables, nuts and reduced intake of refined grains reported a 2 mmHg reduction systolic blood pressure and 0.1 mmHg reduction in diastolic blood pressure^58^. The lack of association between refined grains and hypertension risk, could be due to the much lower content of dietary fiber in refined grains than in whole grains (usually about 70–80% lower), which would reduce any of the above mentioned beneficial effects related to dietary fiber.

These findings have important public health implications as 1.28 billion people globally have hypertension^1^. Based on the results from the US studies that reported the distribution of cases per category, we estimated that 5.6% of cases in one study^15^ and between 9.6 and 10.5% of cases in three studies^14,16,17^ could have been prevented if the study populations moved their whole grain consumption to the highest category of intake. This suggests a sizeable proportion of cases potentially may be preventable by increasing whole grain consumption, however, this would vary based on the level of whole grain consumption in the population of interest. The current recommendations for whole grain consumption in the Nordic countries is in the range of 75–90 g/day^59,60^. In the current meta-analysis, some further reduction in risk was observed with higher intakes up to 400 g/day, however, only one of the included studies reported such a high whole grain intake^22^, thus further studies are needed to clarify the association at very high intakes. The inverse association between whole grain intake and hypertension is in line with previous meta-analyses that reported reductions in risk of cardiovascular disease with higher whole grain consumption, and suggests a potential pathway through which whole grains may reduce cardiovascular risk may be reduced risk of hypertension^9,44^.

In conclusion, these results provide further evidence that a high whole grain intake is associated with reduced risk of hypertension and support dietary recommendations to increase whole grain intake in the general population. There was little evidence of an association between refined grain consumption and hypertension risk.

## Electronic supplementary material

Below is the link to the electronic supplementary material.

Supplementary Material 1

## Data Availability

Data is provided within the manuscript or supplementary information files.
