# Supplementary material for: Whole grain and refined grain consumption and the risk of hypertension: a systematic review and meta-analysis of prospective studies
Source: Sci Rep. 2025 Jul 1;15:21447. doi: 10.1038/s41598-025-05197-5 (PMC12214891; doi:10.1038/s41598-025-05197-5)
Supplement: Supplementary file 1 — Supplementary Material 1 [file 41598_2025_5197_MOESM1_ESM.pdf]

Supplementary information for

Aune D, Metoudi M, Sadler I, Kassam S. Whole grain and refined grain consumption and the risk of hypertension: a systematic review and meta-analysis of prospective studies. Scientific Reports 2025 https://doi.org/10.1038/s41598-025-05197-5

Search strategy

1. Pubmed:

1) ("Edible Grain"[Mesh] OR "Whole Grains"[Mesh] OR “refined grains” [tiab] OR "Zea mays"[Mesh] OR "Triticum"[Mesh] OR "Fagopyrum"[Mesh] OR "Millets"[Mesh] OR "Amaranthus"[Mesh] OR "Chenopodium quinoa"[Mesh] OR "Carya"[Mesh] OR wheat[tiab] OR bread[tiab] OR rice[tiab] OR rye[tiab] OR spelt[tiab] OR barley [tiab] OR corn[tiab] OR maize[tiab] OR quinoa[tiab] OR buckwheat[tiab] OR millets[tiab] OR amaranthus[tiab] OR "Nuts"[Mesh] OR "Arachis"[Mesh] OR "Juglans"[Mesh] OR "Prunus dulcis"[Mesh] OR "Anacardium"[Mesh] OR "Bertholletia"[Mesh] OR "Pistacia"[Mesh] OR Nuts[tiab] OR Peanut[tiab] OR walnut[tiab] OR almond[tiab] OR cashew[tiab] OR pecan[tiab] OR “Brazil nut”[tiab] OR pistachio[tiab] OR "Plant Proteins, Dietary"[Mesh] OR "Vigna"[Mesh] OR "Vicia faba"[Mesh] "Soy Foods"[Mesh] OR "Soybean Proteins"[Mesh] OR Legumes[tiab] OR beans[tiab] OR Lentil[tiab] OR chickpea[tiab] OR Tofu[tiab] OR soya[tiab] OR soy[tiab] OR peas[tiab] OR "Food"[Mesh])

2) ("Hypertension"[Mesh] OR Hypertension[tiab])

3) (cohort OR prospective OR longitudinal OR “case-control” OR “cross-sectional” OR "hazard ratio" OR "relative risk" OR "odds ratio" OR "risk ratio" OR "incidence rate ratio" OR trials OR "Epidemiologic Studies"[Mesh])

4) 1 AND 2 AND 3

2. Embase

1) ‘whole grain*’:ab,ti OR 'wheat':ab,ti OR bread:ab,ti OR rice:ab,ti OR rye:ab,ti OR spelt:ab,ti OR barley:ab,ti OR corn:ab,ti OR maize:ab,ti OR ‘Chenopodium quinoa’:ab,ti OR buckwheat:ab,ti OR millet:ab,ti OR ‘refined grains’:ab,ti OR nut:ab,ti OR nuts:ab,ti OR peanut*:ab,ti OR almond*:ab,ti OR hazelnut*:ab,ti OR ‘pine nut*’:ab,ti OR ‘cashew nut*’:ab,ti OR pecan:ab,ti OR “Brazil nut*”:ab,ti OR pistachio*:ab,ti OR walnut*:ab,ti OR legumes:ab,ti OR beans:ab,ti OR lentil:ab,ti OR chickpea:ab,ti OR tofu:ab,ti OR soya:ab,ti OR soy:ab,ti OR peas:ab,ti

2) 'grain'/exp OR 'bran'/exp OR 'food grain'/exp OR 'nut'/exp

3) 'hypertension':ab.ti

4) 'hypertension'/exp

5) 'clinical study'/exp

6) cohort OR prospective OR longitudinal OR 'case-control' OR 'cross-sectional' OR 'hazard ratio' OR 'relative risk' OR 'odds ratio' OR 'risk ratio' OR 'incidence rate ratio' OR trials

7) 1 OR 2

8) 3 OR 4

9) 5 OR 6

10) 7 AND 8 AND 9

Supplementary Table 1. List of excluded studies and exclusion reasons

Exclusion reason	Reference number
Abstract	(1)
Cross-sectional study	(2-12)
Meta-analysis	(13)
Not relevant exposure	(14;15)
Not relevant outcome	(16-39)
Review	(40-43)
Unspecific exposure	(44)

Reference List

1. Kirwan J, Malin S, Scelsi A et al. A whole grain diet reduces blood pressure in overweight/obese adults; a randomized control trial. FASEB J 2014;28.
2. Esmaillzadeh A, Mirmiran P, Azizi F. Whole-grain consumption and the metabolic syndrome: a favorable association in Tehranian adults. Eur J Clin Nutr 2005;59:353-62.
3. Vernay M, Aidara M, Salanave B et al. Diet and blood pressure in 18-74-year-old adults: the French Nutrition and Health Survey (ENNS, 2006-2007). J Hypertens 2012;30:1920-7.
4. Song S, Lee JE, Song WO, Paik HY, Song Y. Carbohydrate intake and refined-grain consumption are associated with metabolic syndrome in the Korean adult population. J Acad Nutr Diet 2014;114:54-62.
5. Ponce-Martinez X, Colin-Ramirez E, Sanchez-Puerto P et al. Bread Consumption Is Associated with Elevated Blood Pressure among Adults Living in Mexico City - A Sub-Analysis of the Tlalpan 2020 Study. Nutrients 2018;10.
6. Mellendick K, Shanahan L, Wideman L, Calkins S, Keane S, Lovelady C. Diets Rich in Fruits and Vegetables Are Associated with Lower Cardiovascular Disease Risk in Adolescents. Nutrients 2018;10.
7. Zhou L, Feng Y, Yang Y et al. Diet behaviours and hypertension in US adults: the National Health and Nutrition Examination Survey 2013-2014. J Hypertens 2019;37:1230-8.
8. Foscolou A, D'Cunha NM, Naumovski N et al. The Association between Whole Grain Products Consumption and Successful Aging: A Combined Analysis of MEDIS and ATTICA Epidemiological Studies. Nutrients 2019;11.
9. Fulgoni VL, III, Brauchla M, Fleige L, Chu Y. Association of whole-grain and dietary fiber intake with cardiometabolic risk in children and adolescents. Nutr Health 2020;26:243-51.
10. Lanuza F, Zamora-Ros R, Hidalgo-Liberona N, Andres-Lacueva C, Merono T. Wholegrain Consumption and Risk Factors for Cardiorenal Metabolic Diseases in Chile: A Cross-Sectional Analysis of 2016-2017 Health National Survey. Nutrients 2020;12.
11. Tozivepi SN, Takawira S, Chikaka E, Mundagowa P, Chadambuka EM, Mukora-Mutseyekwa F. The Nexus Between Adherence to Recommended Lifestyle Behaviors and Blood Pressure Control in Hypertensive Patients at Mutare Provincial Hospital, Zimbabwe: A Cross-Sectional Study. Patient Prefer Adherence 2021;15:1027-37.

12. Tan D, Sutanto CN, Lin JWX, Toh DWK, Le KA, Kim JE. Measures of carbohydrate quality and their association with diet quality and cardiometabolic health outcomes in Singapore middle-aged and older adults. Nutr Metab Cardiovasc Dis 2023;33:778-88.
13. Saneei P, Larijani B, Esmaillzadeh A. Rice consumption, incidence of chronic diseases and risk of mortality: meta-analysis of cohort studies. Public Health Nutr 2017;20:233-44.
14. Ahn Y, Park SJ, Kwack HK, Kim MK, Ko KP, Kim SS. Rice-eating pattern and the risk of metabolic syndrome especially waist circumference in Korean Genome and Epidemiology Study (KoGES). BMC Public Health 2013;13:61.
15. Riseberg E, Lopez-Cepero A, Mangano KM, Tucker KL, Mattei J. Specific Dietary Protein Sources Are Associated with Cardiometabolic Risk Factors in the Boston Puerto Rican Health Study. J Acad Nutr Diet 2022;122:298-308.
16. He J, Klag MJ, Whelton PK et al. Oats and buckwheat intakes and cardiovascular disease risk factors in an ethnic minority of China. Am J Clin Nutr 1995;61:366-72.
17. Cicero AFG, Fogacci F, Veronesi M et al. Short-Term Hemodynamic Effects of Modern Wheat Products Substitution in Diet with Ancient Wheat Products: A Cross-Over, Randomized Clinical Trial. Nutrients 2018;10.
18. Liu X, Liao X, Gan W et al. Inverse Relationship Between Coarse Food Grain Intake and Blood Pressure Among Young Chinese Adults. Am J Hypertens 2019;32:402-8.
19. Damsgaard CT, Biltoft-Jensen A, Tetens I et al. Whole-Grain Intake, Reflected by Dietary Records and Biomarkers, Is Inversely Associated with Circulating Insulin and Other Cardiometabolic Markers in 8- to 11-Year-Old Children. J Nutr 2017;147:816-24.
20. Katcher HI, Legro RS, Kunselman AR et al. The effects of a whole grain-enriched hypocaloric diet on cardiovascular disease risk factors in men and women with metabolic syndrome. Am J Clin Nutr 2008;87:79-90.
21. Lankinen M, Kolehmainen M, Jaaskelainen T et al. Effects of whole grain, fish and bilberries on serum metabolic profile and lipid transfer protein activities: a randomized trial (Sysdimet). PLoS One 2014;9:e90352.
22. Bahadoran Z, Mirmiran P, Delshad H, Azizi F. White rice consumption is a risk factor for metabolic syndrome in Tehrani adults: a prospective approach in Tehran Lipid and Glucose Study. Arch Iran Med 2014;17:435-40.
23. Song S, Young PH, Song WO, Song Y. Metabolic syndrome risk factors are associated with white rice intake in Korean adolescent girls and boys. Br J Nutr 2015;113:479-87.
24. Kirwan JP, Malin SK, Scelsi AR et al. A Whole-Grain Diet Reduces Cardiovascular Risk Factors in Overweight and Obese Adults: A Randomized Controlled Trial. J Nutr 2016;146:2244-51.
25. Li L, Lietz G, Seal C. Buckwheat and CVD Risk Markers: A Systematic Review and Meta-Analysis. Nutrients 2018;10.
26. Ruggiero E, Bonaccio M, Di Castelnuovo A et al. Consumption of whole grain food and its determinants in a general Italian population: Results from the INHES study. Nutr Metab Cardiovasc Dis 2019;29:611-20.
27. Vedamanickam R, Anandan P, Bupesh G, Vasanth S. Study of millet and non-millet diet on diabetics and associated metabolic syndrome. Biomedicine 2020;40:55-8.
28. Matsuoka T, Hosomi K, Park J et al. Relationships between barley consumption and gut microbiome characteristics in a healthy Japanese population: a cross-sectional study. BMC Nutr 2022;8:23.

29. Davy BM, Melby CL, Beske SD, Ho RC, Davrath LR, Davy KP. Oat consumption does not affect resting casual and ambulatory 24-h arterial blood pressure in men with high-normal blood pressure to stage I hypertension. J Nutr 2002;132:394-8.
30. Keenan JM, Pins JJ, Frazel C, Moran A, Turnquist L. Oat ingestion reduces systolic and diastolic blood pressure in patients with mild or borderline hypertension: a pilot trial. J Fam Pract 2002;51:369.
31. Hallfrisch J, Scholfield DJ, Behall KM. Blood pressure reduced by whole grain diet containing barley or whole wheat and brown rice in moderately hypercholesterolemic men. Nutr Res 2003;23:1631-42.
32. Son JT, Lee E. Effects of the amount of rice in meals on postprandial blood pressure in older people with postprandial hypotension: a within-subjects design. J Clin Nurs 2015;24:2277-85.
33. Meydani M, Thomas M, Barnett JB et al. Short term consumption of whole grain foods independent of weight loss does not affect surrogate markers of cvd. FASEB J 2016;30.
34. Li Y, Mao R, Xu M, Wang J. Effects of Avena nuda L. on chinese patients with hypertension associated with type 2 diabetes mellitus: Secondary analysis of a randomized clinical trial. Ann Nutr Metab 2017;71:901.
35. Leao LSCS, Aquino LA, Dias JF, Koifman RJ. Addition of oat bran reduces HDL-C and does not potentialize effect of a low-calorie diet on remission of metabolic syndrome: A pragmatic, randomized, controlled, open-label nutritional trial. Nutrition 2019;65:126-30.
36. Xue Y, Cui L, Qi J et al. The effect of dietary fiber (oat bran) supplement on blood pressure in patients with essential hypertension: A randomized controlled trial. Nutr Metab Cardiovasc Dis 2021;31:2458-70.
37. Harris JK, West SG, Vanden Heuvel JP et al. Effects of whole and refined grains in a weight-loss diet on markers of metabolic syndrome in individuals with increased waist circumference: a randomized controlled-feeding trial. Am J Clin Nutr 2014;100:577-86.
38. Pins JJ, Geleva D, Keenan JM, Frazel C, O'Connor PJ, Cherney LM. Do whole-grain oat cereals reduce the need for antihypertensive medications and improve blood pressure control? J Fam Pract 2002;51:353-9.
39. Xi H, Zhou W, Niu Y et al. Effect of Oat Consumption on Blood Pressure: A Systematic Review and Meta-Analysis of Randomized Controlled Trials. J Acad Nutr Diet 2023;123:809-23.
40. Harris KA, Kris-Etherton PM. Effects of whole grains on coronary heart disease risk. Curr Atheroscler Rep 2010;12:368-76.
41. Williams PG. The benefits of breakfast cereal consumption: a systematic review of the evidence base. Adv Nutr 2014;5:636S-73S.
42. Priebe MG, McMonagle JR. Effects of Ready-to-Eat-Cereals on Key Nutritional and Health Outcomes: A Systematic Review. PLoS One 2016;11:e0164931.
43. Liska DJ, Dioum E, Chu Y, Mah E. Narrative Review on the Effects of Oat and Sprouted Oat Components on Blood Pressure. Nutrients 2022;14.
44. Liu X, Lai H, Mi B, Qi X, Gan W, Du H. Associations of Coarse Grain Intake with Undiagnosed Hypertension among Chinese Adults: Results from the China Kadoorie Biobank. Nutrients 2020;12.

Supplementary Table 2. Study characteristics of included studies

Author, publication year, country/region	Study name	Follow-up period	Study size, sex, age, number of cases	Dietary assessment method	Exposure	Quantity	RRs (95% CIs)	Adjustment for confounders
Steffen LM et al, 2005, USA	CARDIA Study	1985-2000, 15 years follow-up	4304 men and women, age 18-30 years: 997 elevated blood pressure cases	Validated diet history	Whole grains<br><br><br><br><br>Refined grains	<0.4 times/d<br>0.4-0.7<br>0.7-1.2<br>1.2-1.9<br>>1.9<br><1.8 times/d<br>1.8-2.4<br>2.4-3.1<br>3.1-4.3<br>>4.3	1.00<br>1.00 (0.83-1.20)<br>0.89 (0.78-1.18)<br>0.82 (0.66-1.02)<br>0.83 (0.67-1.03)<br>1.00<br>0.84 (0.68-1.03)<br>0.96 (0.78-1.18)<br>0.98 (0.79-1.21)<br>0.87 (0.68-1.12)	Age, sex, race, center, energy intake, education, physical activity, alcohol intake, smoking, vitamin intake
Wang L et al, 2007, USA	Women's Health Study	1992-2004, 10 years follow-up	28926 women, age 45 years: 8722 hypertension cases	Validated FFQ	Whole grains<br><br><br><br><br>Refined grains	0.21 serv/d<br>0.64<br>1.13<br>1.64<br>3.07<br>0.76 serv/d<br>1.33<br>1.85<br>2.55<br>4.06	1.00<br>0.96 (0.89-1.03)<br>0.95 (0.88-1.02)<br>0.92 (0.85-0.99)<br>0.89 (0.82-0.97)<br>1.00<br>0.97 (0.90-1.04)<br>0.94 (0.87-1.01)<br>0.99 (0.91-1.07)<br>0.97 (0.89-1.06)	Age, race, total energy, randomized treatment assignment, smoking, alcohol, exercise, menopausal status, hormone use, multivitamin use, FH - MI, BMI, diabetes, hypercholesterolemia, fruit and vegetables, meats, dairy products
Flint AJ et al, 2009, USA	Health Professionals Follow-up Study	1986-2004, 18 years follow-up	31684 men, age 40-75 years: 9227 hypertension cases	Validated FFQ	Whole grains	3.3 g/d<br>9.8<br>17.1<br>26.9<br>46.0	1.00<br>0.94 (0.88-1.01)<br>0.89 (0.83-0.95)<br>0.89 (0.84-0.96)<br>0.81 (0.75-0.87)	Age, energy, FH - CHD, FH - hypertension, smoking, alcohol, marital status, profession, height,

								fruit and vegetables, sodium, physical activity, multivitamin use, cholesterol screening
Kochar J et al, 2012, USA	Physicians' Health Study I	1981-1983 - NA, 16.3 years follow-up	13368 men, age 39.7-85.9 years: 5181/3132 hypertension cases (whole grain/refined grain cereals)	FFQ	Whole grain cereals<br><br><br><br>Refined grain cereals	0 serv/wk<br><1<br>2-6<br>≥7<br>0 serv/wk<br><1<br>2-6<br>≥7	1.00<br>0.89 (0.82-0.97)<br>0.87 (0.81-0.94)<br>0.80 (0.74-0.86)<br>1.00<br>0.91 (0.80-1.03)<br>0.86 (0.76-0.98)<br>0.86 (0.74-1.00)	Age, smoking status, BMI, alcohol, fruit and vegetables, physical activity, diabetes mellitus
Shi Z et al, 2012, China	Jiangsu Nutrition Study	2002-2007, 5 years follow-up	1231 men and women, age ≥20 years: 683 cases	FFQ, 33 items	Rice	0-200 g/d<br>201-400<br>≥401	1.00<br>0.68 (0.43-1.08)<br>0.58 (0.36-0.93)	Age, sex, baseline systolic blood pressure, smoking, alcohol drinking, active commuting, leisure-time physical activity, education, occupation, energy intake
Weng L et al, 2013, USA	Atherosclerosis Risk in Communities (ARIC) Study	1987-1998, 9 years follow-up	9913 men and women, age 45-64 years: 1663 hypertension cases	Validated FFQ, 66 items	Whole grains<br><br><br><br><br>Refined grains	0.1 serv/d<br>0.5<br>1.0<br>1.5<br>3.0<br>0.5 serv/d<br>1.5<br>2.0<br>3.0<br>5.0	1.00<br>1.04 (0.93-1.17)<br>1.03 (0.91-1.16)<br>1.00 (0.89-1.13)<br>0.99 (0.87-1.12)<br>1.00<br>1.04 (0.92-1.16)<br>0.99 (0.88-1.12)<br>1.03 (0.91-1.17)<br>1.00 (0.87-1.15)	Age, sex, race, education, center, energy intake, added salt, physical activity, smoking

Lelong H et al, 2017, France	NutriNet-Sante	2009-2015, 3.4 years follow-up	80426 men and women, age ≥18 years: 2413 hypertension cases	Validated 24-hour recall	Whole grains	0 g/d<br>9<br>31<br>102	1.00<br>0.97 (0.85-1.12)<br>0.92 (0.83-1.02)<br>0.84 (0.76-0.93)	Age, sex, smoking status, alcohol, BMI, physical activity, education, total energy, FH - hypertension
Quinteiros Fidalgo AS, 2018, Switzerland	The CoLaus Study	2009-2017, 5.25 years follow-up	2079 men and women, age 40-80 years: 370 hypertension cases	Validated FFQ, 97 food items	Whole grains	2.6/0.0 g/d w/m<br>23.4/12.5<br>51.8/41.0<br>123.2/96.7	1.00<br>1.09 (0.80-1.49)<br>0.85 (0.61-1.18)<br>0.94 (0.67-1.33)	Age, sex, BMI, education, sedentariness, diabetes, total energy intake
Kashino I et al, 2020, Japan	Furukawa Nutrition and Health Study	2012-2015, 3 years follow-up 2013-2016, 3 years follow-up	944 men and women, age 19-68 years: 86 hypertension cases	Diet history questionnaire	Whole grains	Never<br>Rarely<br>Sometimes or always	1.00<br>1.02 (0.57-1.83)<br>0.36 (0.16-0.83)	Age, sex, smoking, alcohol consumption, physical activity, shift work, total energy intake, sodium, vegetables, fruits, pulses, meat, dairy, soft drinks, rice, bread, noodles
Xu ZH et al, 2024, China	China Health and Nutrition Survey	1997-2015, 7.0 years follow-up	10973 men and women, age ≥18 years (mean 40.3): 3733 hypertension cases	24 hour recalls	Whole grains<br><br><br><br>Whole grain wheat<br><br><br><br>Whole grain maize	<103.0 g/d<br>103.0-<250.5<br>250.5-<546.6<br>≥546.6<br>1<br>2<br>3<br>4<br>1<br>2<br>3	1.00<br>0.52 (0.47-0.57)<br>0.46 (0.42-0.51)<br>0.35 (0.31-0.38)<br>1.00<br>0.53 (0.48-0.58)<br>0.46 (0.41-0.50)<br>0.35 (0.32-0.39)<br>1.00<br>0.50 (0.42-0.60)<br>0.53 (0.44-0.63)	Age, sex, survey year, BMI, smoking status, drinking status, systolic blood pressure, diastolic blood pressure, education, occupation, region, urban/rural residency, physical activity, total energy

						4	0.50 (0.42-0.59)	
					Whole grain millet	1<br>2<br>3<br>4	1.00<br>0.44 (0.36-0.55)<br>0.48 (0.38-0.59)<br>0.38 (0.30-0.48)	

BMI=body mass index, CHD=coronary heart disease, FH=family history, MI=myocardial infarction

Supplementary Table 3. Definitions of whole grains across studies

Author, publication year, country/region	Study name	Whole grain and refined grain definition
Steffen LM et al, 2005, USA	CARDIA Study	Not further described
Wang L et al, 2007, USA	Women's Health Study	Whole grain foods included dark bread, whole grain breakfast cereal, popcorn, cooked oatmeal, wheat germ, brown rice, bran and other grains. Breakfast cereals with ≥25% whole grain or bran content by weight were considered as whole grains.<br><br>Refined grains included sweet rolls and cakes or desserts, white bread, pasta, English muffins, muffins or biscuits, refined grain breakfast cereal, white rice, pancakes, waffles, pizza
Flint AJ et al, 2009, USA	Health Professionals Follow-up Study	Intakes of whole grains, bran and germ were calculated by determining the whole grain content of each grain food according to the dry weight of its whole grain ingredients.
Kochar J et al, 2012, USA	Physicians' Health Study I	Breakfast cereals with at least 25% of oat or bran were classified as whole grain.
Shi Z et al, 2012, China	Jiangsu Nutrition Study	Rice
Weng L et al, 2013, USA	Atherosclerosis Risk in Communities (ARIC) Study	Not further described
Lelong H et al, 2017, France	NutriNet-Sante	Not further described
Quinteiros Fidalgo AS, 2018, Switzerland	The CoLaus Study	Not further described
Kashino I et al, 2020, Japan	Furukawa Nutrition and Health Study	Brown rice, germinated rice, wheat/millet mix with rice
Xu ZH et al, 2024, China	China Health and Nutrition Survey	Flours made from wheat, rye, oats, barley, maize, millet sorghum, buckwheat, whole corn and corn flour and their associated products (excluding popcorn).

Supplementary Table 4. Modified Newcastle-Ottawa Scale (NOS) assessment of the included cohort studies

Author, Year	Selection		Comparability		Outcome assessment			Total	[a]Score
	Selection of non-exposed cohort	Exposure ascertainment	Demonstration of outcome not present at start	Comparability (0.25 points for each adjustment)	Outcome assessment	Long enough follow-up (>3 years)	Adequacy of follow-up (< 10% loss)		
Steffen, 2005	1	1	1	2	1	1	0	7	High
Wang, 2007	1	1	1	2	0	1	0	6	Medium
Flint, 2009	1	1	1	2	0	1	0	6	Medium
Kochar, 2012	1	0	1	1.75	0	1	0	3.75	Medium
Shi, 2012	1	1	0	2	1	1	0	6	Medium
Weng, 2013	1	1	1	2	1	1	0	7	High
Lelong, 2017	1	1	1	1.75	0	1	0	6	Medium
Quinteiros Fidalgo, 2018	1	1	1	1.75	1	1	0	6.75	High
Kashino, 2020	1	1	1	2	1	1	0	7	High
Xu, 2024	1	1	1	2	1	1	0	7	High

[a]Overall quality score was assessed according to a categorical score domain of low (0-3 points), medium (>3-6 points) and high (>6-8 points).

Supplementary Table 5. RRs (95% CIs) from nonlinear dose-response analysis of whole grains and hypertension

g/d	RR (95% CI)	E-value (lower CI)
0	1.00	
50	0.90 (0.85-0.97)	1.46 (1.21)
100	0.85 (0.79-0.91)	1.63 (1.43)
150	0.81 (0.75-0.88)	1.77 (1.53)
200	0.78 (0.68-0.88)	1.88 (1.53)
250	0.74 (0.62-0.88)	2.04 (1.53)
300	0.71 (0.56-0.89)	2.17 (1.50)
350	0.67 (0.51-0.89)	2.35 (1.50)
400	0.64 (0.46-0.90)	2.50 (1.46)
$p_{nonlinearity}$	0.31	

Supplementary Table 6. RRs (95% CIs) from nonlinear dose-response analysis of refined grains and hypertension

g/d	RR (95% CI)
0	1.00
30	0.97 (0.93-1.01)
60	0.94 (0.87-1.02)
90	0.93 (0.85-1.02)
120	0.93 (0.85-1.02)
150	0.94 (0.86-1.03)
$p_{nonlinearity}$	0.21

Supplementary Table 7. Whole grains and hypertension, subgroup analyses (per 90 g/d)

Subgroup		n	Summary RR (95% CI)	I^2	$p_{heterogeneity}$	$p_{heterogeneity}$
All		8	0.86 (0.82-0.90)	63.3	0.008	
Sex	Men & women	5	0.87 (0.84-0.91)	22.3	0.27	0.23/0.59[1]
	Men	2	0.76 (0.60-0.97)	90.4	0.001	
	Women	1	0.89 (0.82-0.97)			
Duration of follow-up	<10 years	4	0.87 (0.84-0.90)	15.2	0.32	0.21
	10 years	4	0.80 (0.71-0.90)	78.4	0.003	
Geographic location	America	5	0.83 (0.75-0.92)	78.5	0.001	0.90
	Europe	2	0.87 (0.80-0.94)	0	0.70	
	Asia	1	0.86 (0.85-0.88)			
Number of cases	<1000	2	0.82 (0.65-1.02)	28.9	0.24	0.76
	≥1000	6	0.86 (0.82-0.90)	71.2	0.004	
Study quality	≤3 stars	0				0.45
	>3-6	4	0.83 (0.76-0.90)	76.8	0.005	
	>6-8	4	0.88 (0.81-0.96)	41.6	0.16	
Validated dietary assessment	Yes	6	0.84 (0.76-0.93)	73.5	0.002	0.81
	No	2	0.86 (0.85-0.88)	0	0.66	
Repeated dietary assessment	Yes	4	0.81 (0.71-0.94)	83.5	<0.0001	0.47
	No	4	0.87 (0.83-0.90)	0	0.83	
Adjustment for confounders						
Age	Yes	8	0.86 (0.82-0.90)	63.3	0.008	NC
	No	0				
Education	Yes	5	0.87 (0.84-0.91)	22.3	0.27	0.39
	No	3	0.81 (0.71-0.92)	84.3	0.002	
Family history of hypertension	Yes	2	0.76 (0.59-0.98)	89.7	0.002	0.18
	No	6	0.87 (0.85-0.89)	14.6	0.32	
Smoking	Yes	7	0.85 (0.81-0.90)	68.3	0.004	0.70
	No	1	0.91 (0.70-1.18)			
Alcohol	Yes	6	0.84 (0.80-0.88)	67.5	0.009	0.21
	No	2	0.96 (0.86-1.07)	0	0.67	
BMI	Yes	5	0.86 (0.85-0.88)	0	0.93	0.34
	No	3	0.78 (0.59-1.03)	88.0	<0.001	
Physical activity	Yes	7	0.85 (0.81-0.90)	68.3	0.004	0.70
	No	1	0.91 (0.70-1.18)			
Fruits, vegetables	Yes	3	0.81 (0.71-0.92)	84.3	0.002	0.39
	No	5	0.87 (0.84-0.91)	22.3	0.27	
Red, processed meat	Yes	1	0.89 (0.82-0.97)			0.64
	No	7	0.85 (0.80-0.90)	67.6	0.005	
Dairy products	Yes	1	0.89 (0.82-0.97)			0.64
	No	7	0.85 (0.80-0.90)	67.6	0.005	
Salt	Yes	2	0.80 (0.56-1.16)	93.7	<0.001	0.58
	No	6	0.86 (0.85-0.88)	0	0.78	
Energy intake	Yes	5	0.86 (0.80-0.92)	76.7	0.002	0.88
	No	3	0.85 (0.81-0.90)	0	0.45	

[1] $P_{heterogeneity}$ when excluding studies of men and women combined.

Supplementary Table 8. Evidence grading for whole grains and refined grains and hypertension

Requirements for grading of convincing	Whole grains	Refined grains
Statistically significant and robust association	Statistically significant and robust inverse association both in high vs. low and dose-response analyses. No evidence of nonlinearity in nonlinear dose-response analyses. One study could not be included in the dose-response analysis because of lack of quantification of whole grain intake.	No statistically significant association in high vs. low, linear and nonlinear dose-response analyses.
Evidence from at least two independent cohort studies	9 studies (high vs. low)<br>8 studies (dose-response	5 studies (high vs. low)<br>4 studies (dose-response
No substantial unexplained heterogeneity within or between study types or in different populations relating to the presence or absence of an association, or direction of effect	There is high heterogeneity in the high vs. low analysis, however, this is reduced considerably in the dose-response analysis, to a large extent because of a very high intake in one study resulting in a very strong inverse association, however, when standardized to the same increment the results are comparable with the other studies. The inverse association is observed across most subgroup analyses and there is little evidence of heterogeneity between subgroups.	The heterogeneity is low in both high vs. low and dose-response analyses. Subgroup analyses were not conducted because of a limited number of studies.
Good quality studies to exclude with confidence the possibility that the observed association results from random or systematic error, including confounding, measurement error, and selection bias	No indication of publication bias<br><br>Results persisted in most subgroup analyses. There was no indication of between subgroup heterogeneity with meta-regression analyses.<br><br>No studies corrected for measurement error.<br><br>All studies excluded prevalent hypertension cases at baseline. Exposed and non-exposed participants were selected from the same populations.	No indication of publication bias.<br><br>Subgroup analyses were not conducted because of few studies.<br><br>No studies corrected for measurement error.<br>.<br><br>All studies excluded prevalent hypertension cases at baseline. Exposed and non-exposed participants were selected from the same populations.
Presence of a plausible biological gradient in the	Indication of a dose-response relationship up to 400 g/day, and no evidence of nonlinearity.	There is no evidence of nonlinearity or of an association.

association. Such a gradient need not be linear or even in the same direction across different levels of exposure, so long as this can be explained plausibly		
Strong and plausible experimental evidence, either from human studies or relevant animal models, that typical human exposures can lead to relevant outcomes	Whole grains are important sources of dietary fiber, for which there is strong evidence of a protective effect on weight gain, which is an important risk factor for hypertension.<br><br>There is also some evidence from observational studies that a high whole grain intake reduces weight gain and prevents the development of overweight and obesity, which is a strong risk factor for hypertension. There is evidence from randomized trials that high fiber intake reduces blood pressure, and some indication of similar, but less precise effects for whole grains.	Refined grains have been associated with greater weight gain over time, which would support a potential increased risk of hypertension, but we did not observed any association.
Final grading and justification for overall assessment.	Probable evidence for reduced risk of hypertension with higher intake of whole grains.<br><br>Justification: Based on highly significant associations in high vs. low analysis and in linear and nonlinear dose-response analyses. Results are in general consistent across most subgroups. Moderate heterogeneity, no indication of publication bias, no indication of selection bias. Biologically plausible mechanisms exist.	Limited no conclusion.<br><br>Justification: There is no evidence of an association and the number of studies is limited.

Supplementary Figure 1. Whole grains and hypertension, high vs. low

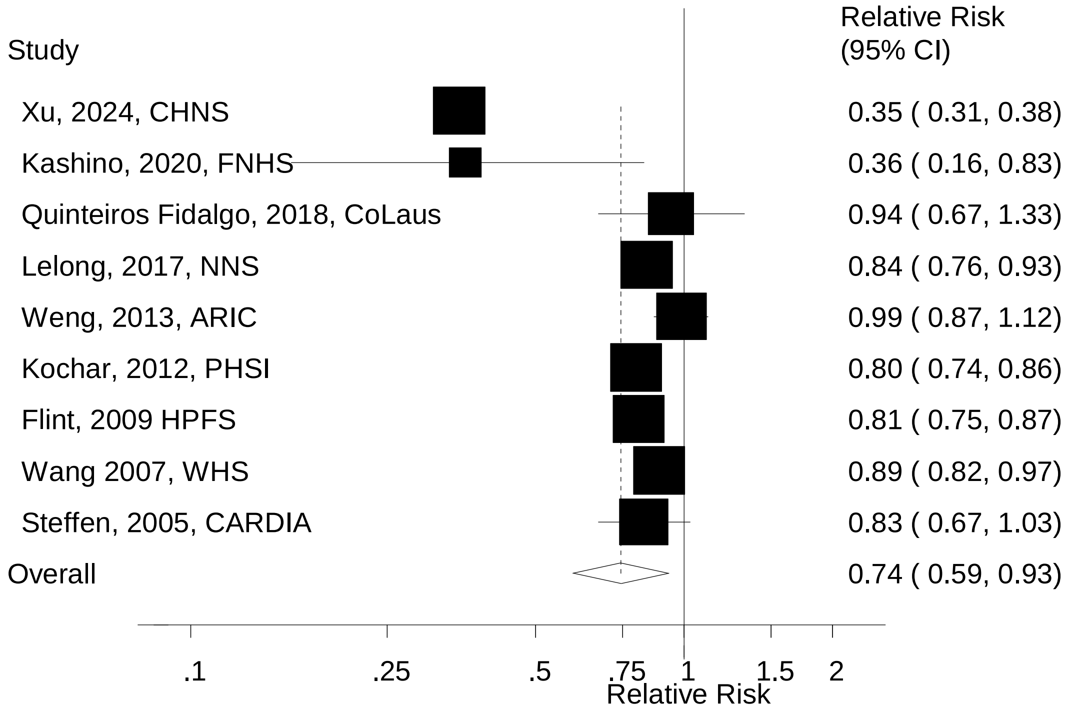

Supplementary Figure 2. Whole grains and hypertension, influence analysis

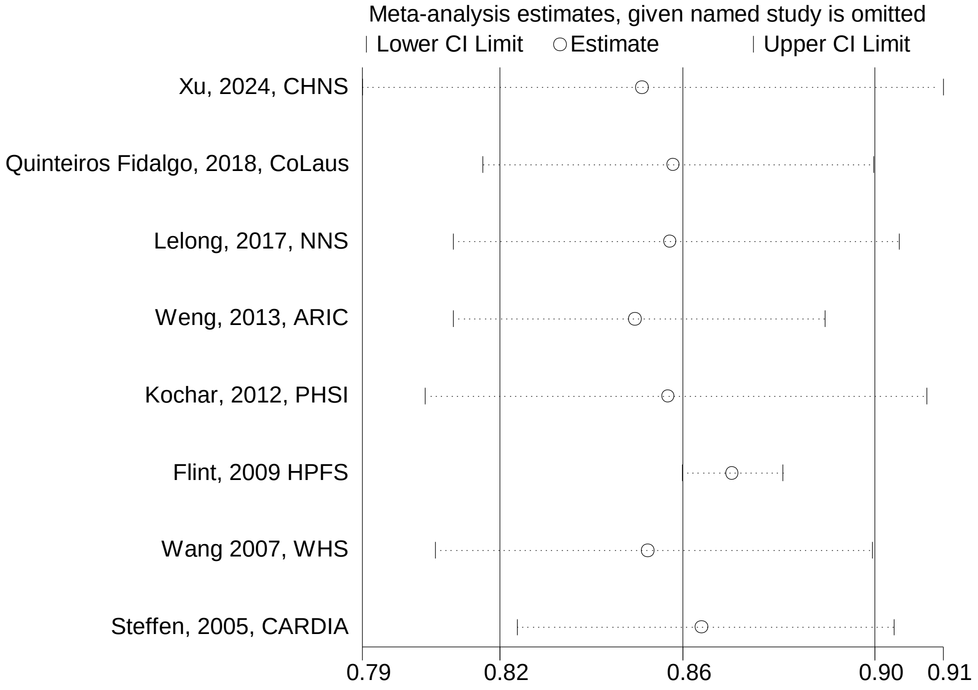

Study omitted	Estimate	[95% Conf.	Interval]
Xu, 2024, CHNS	0.84615898	0.78543681	0.91157556
Quinteiros Fidalgo, 2018, CoLaus	0.85297096	0.81163359	0.89641368
Lelong, 2017, NNS	0.85222191	0.80516374	0.90203041
Weng, 2013, ARIC	0.84459019	0.80519789	0.88590962
Kochar, 2012, PHSI	0.85178208	0.79913855	0.90789354
Flint, 2009 HPFS	0.86574185	0.8548522	0.87677026
Wang 2007, WHS	0.84738618	0.80124021	0.89618987
Steffen, 2005, CARDIA	0.85901123	0.81904411	0.90092868
Combined	0.85509088	0.81538747	0.89672755

Supplementary Figure 3. Whole grains and hypertension, funnel plot

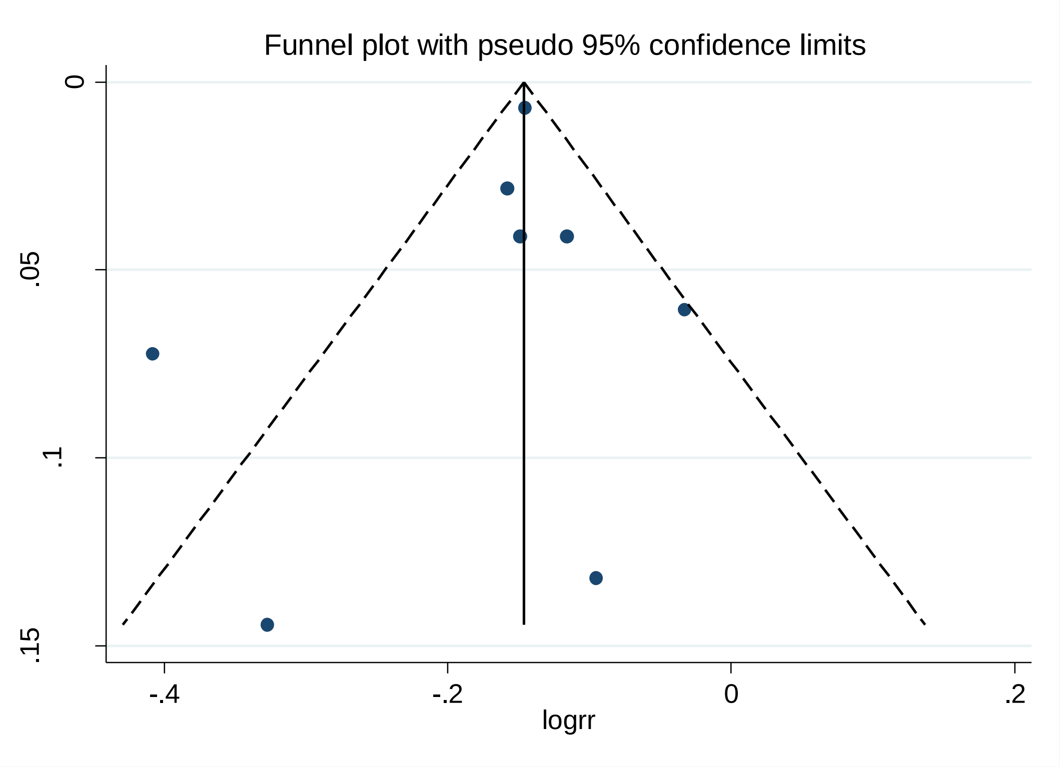

Egger's test, p=0.59
Begg's test, p=0.54

Supplementary Figure 4. Refined grains and hypertension, high vs. low

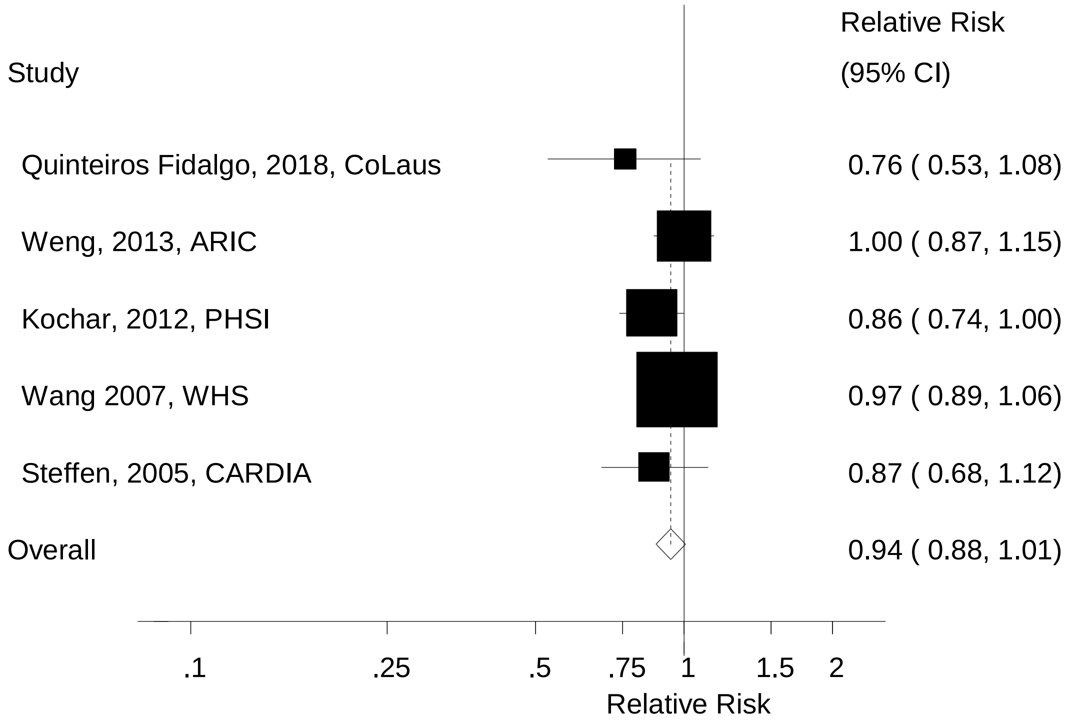

I^2=7.9%, p=0.36

Supplementary Figure 5. Influence analysis of refined grains and hypertension

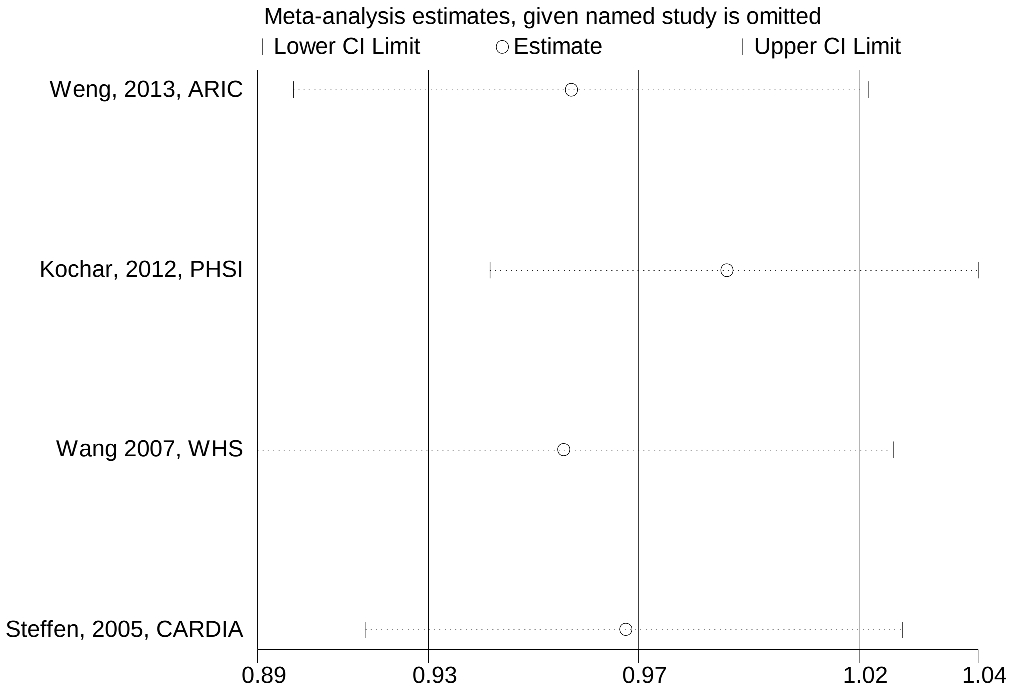

Study omitted	Estimate	[95% Conf.	Interval]
Weng, 2013, ARIC	0.95634866	0.89570117	1.0211025
Kochar, 2012, PHSI	0.99029225	0.93851739	1.0449233
Wang 2007, WHS	0.95467418	0.8878786	1.0264949
Steffen, 2005, CARDIA	0.96821153	0.91144556	1.028513
Combined	0.97088016	0.92511426	1.0189101

Supplementary Figure 6. Funnel plot of refined grains and hypertension

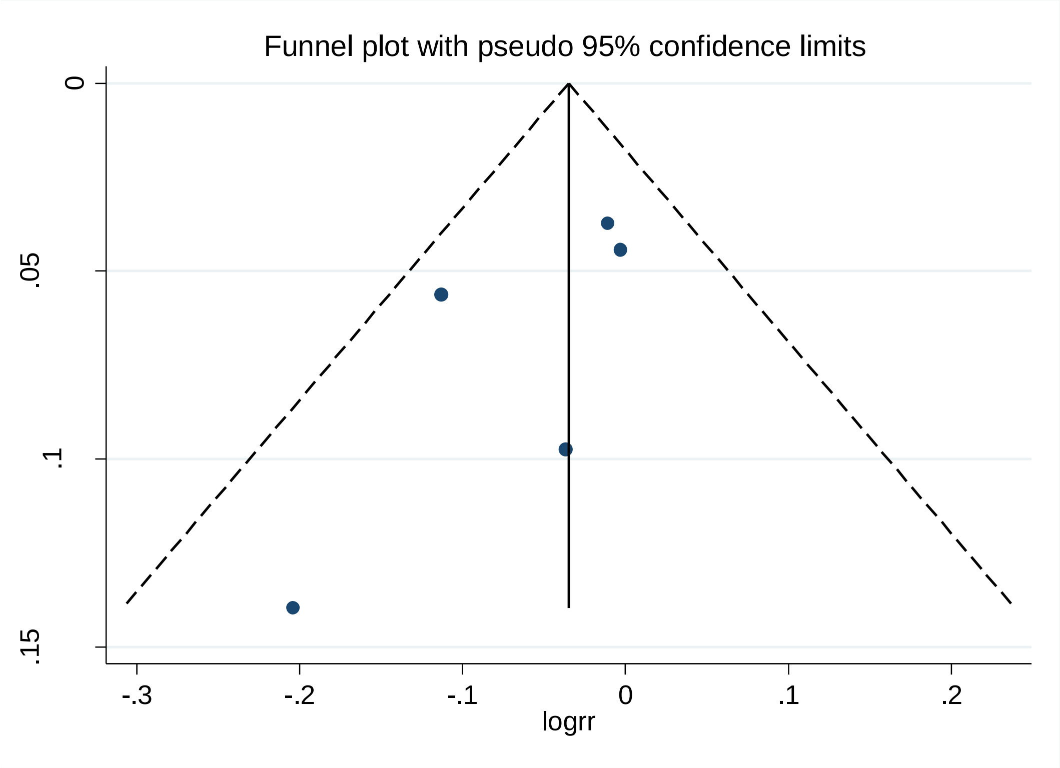

Egger's test, p=0.21
Begg's test, p=0.46
